# Effectiveness of artificial intelligence-assisted colonoscopy in early diagnosis of colorectal cancer: a systematic review

**DOI:** 10.1097/JS9.0000000000000285

**Published:** 2023-03-14

**Authors:** Aashna Mehta, Harendra Kumar, Katia Yazji, Andrew A. Wireko, Jai Sivanandan Nagarajan, Bikona Ghosh, Ahmad Nahas, Luis Morales Ojeda, Ayush Anand, Medha Sharath, Helen Huang, Tulika Garg, Arda Isik

**Affiliations:** a Faculty of Medicine, University of Debrecen, Debrecen, Hungary; b Dow University of Health Sciences, Karachi, Pakistan; c RCSI University of Medicine and Health Sciences, Dublin, Ireland; d Sumy State University, Sumy, Ukraine; e BP Koirala Institute of Health Sciences, Dharan, Nepal; f Dhaka Medical College and Hospital, Dhaka, Bangladesh; g Institute of Urology, University of Southern California, Los Angeles California, USA; h SRM College Hospital and Research Centre Los Angeles; i Bangalore Medical College and Research Institute, Bangalore, Karnataka; j Government Medical College and Hospital, Chandigarh, Punjab, India; k Department of General Surgery, Istanbul Medeniyet University, Istanbul, Turkey

**Keywords:** artificial intelligence, colonoscopy, colorectal cancer, screening

## Abstract

As artificial intelligence (AI)-assisted diagnosis gained immense popularity, it is imperative to consider its utility and efficiency in the early diagnosis of colorectal cancer (CRC), responsible for over 1.8 million cases and 881 000 deaths globally, as reported in 2018. Improved adenoma detection rate, as well as better characterizations of polyps, are significant advantages of AI-assisted colonoscopy (AIC). This systematic review (SR) investigates the effectiveness of AIC in the early diagnosis of CRC as compared to conventional colonoscopy.

**Materials and Methods:**

Electronic databases such as PubMed/Medline, SCOPUS, and Web of Science were reviewed for original studies (randomized controlled trials, observational studies), SRs, and meta-analysis between 2017 and 2022 utilizing Medical Subject Headings terminology in a broad search strategy. All searches were performed and analyzed according to the Preferred Reporting Items for Systematic Reviews and Meta-Analysis methodology and were conducted from November 2022. A data extraction form based on the Cochrane Consumers and Communication Review group’s extraction template for quality assessment and evidence synthesis was used for data extraction. All included studies considered for bias and ethical criteria and provided valuable evidence to answer the research question.

**Results:**

The database search identified 218 studies, including 87 from PubMed, 60 from SCOPUS, and 71 from Web of Science databases. The retrieved studies from the databases were imported to Rayyan software and a duplicate article check was performed, all duplicate articles were removed after careful evaluation of the data. The abstract and full-text screening was performed in accordance with the following eligibility criteria: Strengthening the Reporting of Observational Studies in Epidemiology (STROBE) for observational studies; Preferred Reporting Items for Systematic Reviews and Meta-Analysis for review articles, ENTREQ for narrative studies; and modified JADAD for randomized controlled trials. This yielded 15 studies that met the requirements for this SR and were finally included in the review.

**Conclusion:**

AIC is a safe, highly effective screening tool that can increase the detection rate of adenomas, and polyps resulting in an early diagnosis of CRC in adults when compared to conventional colonoscopy. The results of this SR prompt further large-scale research to investigate the effectiveness in accordance with sex, race, and socioeconomic status, as well as its influence on prognosis and survival rate.

## Introduction

Highlights

- Colorectal cancer, one of the leading cancers is responsible for 881 000 deaths worldwide as of 2018.
- Screening with colonoscopy plays a vital role in early detection as well as prompt management.
- Advent of artificial intelligence in colonoscopy improves adenoma and polyp detection, especially those usually missed by conventional colonoscopy, thereby reducing adenoma miss rate and polyp reduction rate by 50%.
- In addition, one study estimates it to reduce costs associated by colorectal cancer care by 8.2%, with wide implications for patients belonging to different sociodemographics.

Artificial intelligence (AI), one of the most popular innovative solutions in present-day medicine, has emerged as an essential tool in the pattern recognition of images, macroscopic lesions, and histology slides over the last few decades. However, this technology is still far from being fully utilized in a clinical setting, and many studies show that when it is used, there is a real statistical benefit to patient outcomes.

Colonoscopy imaging is one of many fields that can benefit from AI. With over 1.8 million cases and 881 000 deaths from colorectal cancer (CRC) worldwide, there is an enormous burden on healthcare systems. This is exacerbated by an economic burden that can be further reduced by earlier detection of neoplastic lesions^1^. Conventional colonoscopy, one of the most popular screening modalities, although widely used, is limited by the availability of well-trained gastroenterologists to detect early precancerous lesions with a high probability of malignant transformation. A successful early detection is crucial to allow for can involve simple excision for biopsy during the colonoscopy procedure. However, missed lesions can result in delayed diagnosis, and the patient potentially undergoes laborious chemotherapy and radical surgical resection that can severely limit the normal defecation function and ultimately cause the patient’s demise. In addition, a late diagnosis may necessitate complicated and costly surgeries, adjuvant chemotherapy, and long-term follow-ups.

In order to improve CRC care, early diagnosis with continuous improvements in the precision and accuracy of diagnostic tools is required. One such step in this direction is the implementation of AI-assisted colonoscopy (AIC), which has the potential to prevent an estimated 7194 CRC cases and save 290 million US dollars per year, according to microsimulations comparing AI versus non-AI screening for individuals at average risk^2^. In Hungary, for example, the benefits of a well-organized national population-based CRC screening program based on fecal immunochemical testing have a cost-benefit ratio of 8000–8700 Euros per life^3^. It is hypothesized that this could be due to limited access to pharmacists and pharmacies with access to fecal immunochemical testing. Considering this, the population could greatly benefit from organized screening that is based on employing innovative advances and technological solutions such as AIC with the potential of improving diagnostic precision. Similarly, the limited number of well-equipped gastroenterology centers with access to trained specialist gastroenterologists and appropriate colonoscopy equipment causes problems. AIC can help close this gap by lowering the need for specialized knowledge to detect ambiguous results. AI can add a layer of confidence to current tools, allowing for a more confident and accurate diagnosis.

Different polyps and pathologies seen in AIC can be subcharacterized and their prognosis evaluated in real-time using machine learning approaches to improve early computer-aided diagnosis^4^.

Furthermore, further advancements in AI recognition software can help reduce diagnostic time as well as the likelihood of missed diagnosis because a higher detection rate can provide early diagnosis and intervention, potentially improving patient outcomes and survival. To determine its suitability and utility, this systematic review (SR) investigates the effectiveness of AIC in the detection of precancerous lesions compared to conventional colonoscopy, with a special emphasis on its cost-effectiveness.

## Materials and methods

### Research aim and search strategy

**Figure 1:**
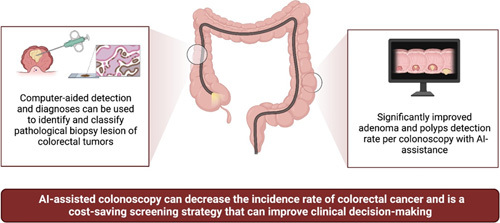
Illustration representing possible benefits of artificial intelligence (AI) and its applications such as computer-aided detection in diagnoses of colorectal cancer.

The studies for the SR were chosen using an adapted Preferred Reporting Items for Systematic Reviews and Meta-Analyses (PRISMA) as shown in the flow diagram in Figure 1 and was registered on PROSPERO ID: CRD42022373188^5^. A preliminary protocol was carried out to guide the literature search. Studies were screened based on the following criteria:

- P (Population): adults (>18) with adenoma, polyp, or colorectal carcinoma.
- I (Intervention): AIC.
- C (Comparison): conventional colonoscopy.
- O (Outcomes): detection of adenomas, polyps, colorectal carcinoma.

**Figure 2:**
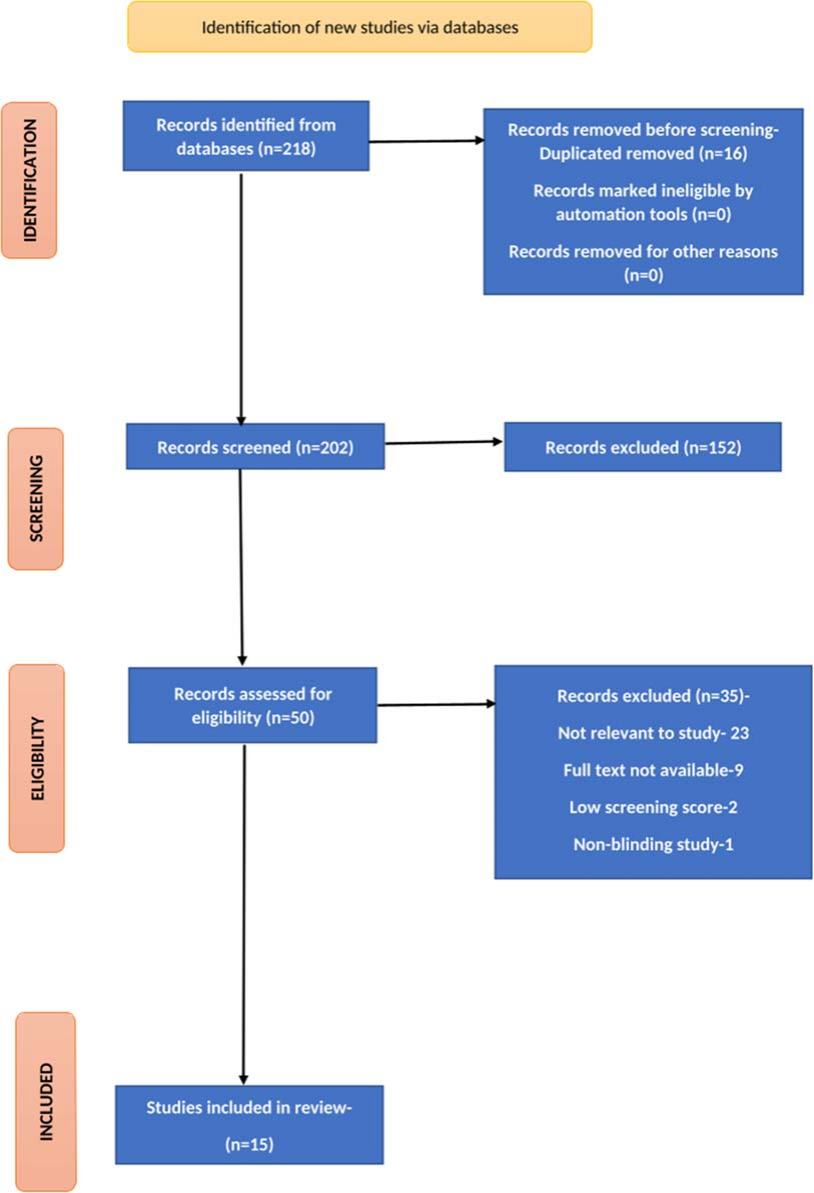
Schematic representation of study selection.

Three databases PubMed/Medline, Web of Science, and SCOPUS were searched for articles from 2018 to 2022 with search equations that included Medical Subject Headings terms (Table 1). The retrieved studies from the databases were imported to Rayyan software and a duplicate article check was performed, all duplicate articles were removed after careful evaluation of the data (Fig. 2).

**Table 1:** Detailed search strategy for PubMed, SCOPUS, WOS.

Database	Equation	Filters
PubMed	(((‘Colonoscopy’[Mesh]) AND ‘Artificial Intelligence’[Mesh]) OR ‘Cost-Benefit Analysis’[Mesh]) AND ‘Colorectal Neoplasms’[Mesh]	2018–2022
Scopus	colonoscopy AND colon cancer OR colorectal neoplasm AND cost-benefit	2018–2022
Web of Science	(((ALL=(AI-assisted colonoscopy)) AND AB=(colorectal neoplasms)) OR AB=(colon cancer)) AND ALL=(cost-benefit)	Last 5 years

AI, artificial intelligence; MESH, Medical Subject Headings.

### Selection criteria

Inclusion and exclusion criteria (Table 2) were established by discussion among the authors to include quality studies for data extraction. Inclusion criteria included original studies, observational studies, SRs, meta-analyses (MAs), and case series specific to AIC in adults over the last 5 years (2018–2022). Exclusion criteria were narrative reviews, editorials, short communications, case studies, as well as scientific articles in languages other than English, and articles for which full text was not retrievable were excluded from this review. In addition, articles on pediatric patients and articles not relevant to the research question were excluded.

**Table 2:** Inclusion and exclusion criteria for study selection.

	Inclusion	Exclusion
Population	Patients with adenoma, polyp, or carcinoma above 18 years or more	A. Animal studies B. Studies not published in English C. Not relevant to study D. Full text not available E. Low screening score F. Nonblinding study
Intervention	Adenoma, polyp, carcinoma detected by AI colonoscopy	
Comparators	Adenoma, polyp, carcinoma detected by conventional colonoscopy	
Study designs	Original studies, observational studies, systematic review, and meta-analysis, case series	Narrative reviews, editorials, short communications, case studies

AI, artificial intelligence.

### Data extraction and management

Data extraction was done by using a standard template based on the Cochrane Consumers and Communication Review group’s extraction template for quality assessment and evidence synthesis. The information extracted in the data list included: authors, database, journal, date of publication, type of article, DOI, original title, full article abstract, applied methodology, and results. Four review authors (A.M., B.G., H.K., and M.S.) screened each abstract, title, or both of the records retrieved and investigated the full text of all potentially relevant records, mapped the records to studies, and classified the studies as included studies or excluded studies. Data were extracted independently by three authors (H.K., A.A., and J.S.), and any discrepancies were discussed and resolved with the other two authors where needed.

### Analysis and synthesis of data

The studies reported in the study were summarized in a narrative fashion. The information was classified into adenoma detection, polyp detection, and false negative rates. Studies containing other information than that about the mentioned groups were included in the miscellaneous category.

### Quality assessment

Different guidelines were used for the quality assessment according to the type of study. The Strengthening the Reporting of Observational Studies in Epidemiology (STROBE) statement provides guidelines for reporting observational studies and assesses the quality of observational studies. PRISMA checklist was used to qualify SRs and MAs, while ENTREQ guideline was followed for narrative reviews. For randomized controlled trials (RCTs), modified JADAD guidelines were followed for assessment. Ethical criteria and bias were evaluated, and all included studies provided important information to answer the research question.

### Evaluation of the studies

Following the abstract screening, full-text screening was carried out on an Excel spreadsheet that included basic data of the study (title, author, year of publication, link to full-text) as well as the score allotted utilizing eligibility criteria. Only studies meeting at least 80% of the checklist requirements were included in the study.

## Results

### Study selection

The database search identified 218 studies, including 87 from PubMed/MEDLINE, 60 from SCOPUS, and 71 from Web of Science databases. The retrieved studies from the databases were imported to Rayyan software and a duplicate article check was performed, all duplicate articles were removed after careful evaluation of the data. Four authors (A.M., B.G., H.K., and M.S.) carried out the title and abstract screening, followed by a full-text screening of the selected articles which were reviewed in accordance with the following eligibility criteria: Strengthening the Reporting of Observational Studies in Epidemiology for observational studies; PRISMA for review articles, ENTREQ for narrative studies; and modified JADAD for RCTs. To ensure the quality of reviewing, each article was reviewed by two authors in abstract and full-text screening. Only articles with an 80% score or better were included in the review. Additionally, we searched the reference list of the relevant articles to identify any relevant papers, which yielded 15 studies that met the requirement for our SR and were finally included in the review (Table 3).

**Table 3:** Studies comparing AI-assisted colonoscopy with conventional colonoscopy on the effectiveness of detecting adenomas and polyps.

References	Country	*N*	Study type	Adenoma detection (AI-assisted)	Adenoma detection× (colonoscopy)	Polyp detection (AI-assisted)	Polyp detection (colonoscopy)
Nazarian *et al*. 6	England	≈29 079	Systematic review and meta-analysis	OR: 1.53, 95% CI: 1.32–1.77; *P*<0.001	NA	OR: 1.75, 95% CI: 1.56–1.96; *P*<0.001	NA
Repici *et al*. 7	Italy	685	Randomized controlled trial	54.80%	40.40%	NA	NA
Repici *et al*. 8	Italy	660	Randomized controlled trial	53.30%	44.50%	NA	NA
Spadaccini *et al*. 9	Italy and USA	34,445	Systematic review and meta-analysis	7.4% higher with CADe (OR: 1.78, 95% CI: 1.44–2.18)	4.4% higher with chromoendoscopy (1.22; 1.08–1.39), and 4.1% higher with increased mucosal visualization systems (1.16, 1.04–1.28)	NA	NA
Areia *et al*. 2	USA	100 000	A modeling study	44.2%	48.9%	NA	NA
Aziz *et al*. 10	USA	2815	Systematic review with meta-analysis	32.90%	20.80%	43.00%	27.80%
Deliwala *et al*. 11	USA	4996	Meta-analysis	OR=1.77	OR=1	OR=1.91	1
Glissen Brown *et al*. 12	USA	234	Randomized controlled trial	79.88%	68.75%	79.30%	66.29
Wallace *et al*. 13	Italy, UK, USA	230	Randomized controlled trial	29 (25.0%) of 116 patients	52 (45.6%) of 114	33 (28.5%) of 116 patients	55 (48.3%) of 114
Wang *et al*. 14	China	382	Randomized controlled trial	42.39%	35.68%	63.59%	55.14%
Yao *et al*. 15	China	1076	Randomized controlled trial	CADe 21.27% (95% CI 16.37–26.17), CAQ 24.54% (95% CI: 19.39–29.68)	14.76% (95% CI: 10.54–18.98)	55.60% (95% CI 49.65–61.55), 53.53% (95% CI: 47.57–59.49)	41.70% (95% CI: 35.83–47.57)

AI, artificial intelligence; *N*, number of study participants; CAQ, computer-aided quality; OR, odd ratios; CADe, computer-aided detection; NA, nonavailability.

Colonoscopy with AI detects more precancerous lesions and CRC than conventional colonoscopy. Eleven studies compared the adenoma and polyp detection in the colon between conventional colonoscopy and AIC. Six of the studies were RCT, four were SR/MAs, and one was a modeling study. The technologies reported in the studies were computer-aided detection (CADe), colonoscopy with computer-aided quality improvement, and DCNN-based AI systems.

The 11 studies looked at adenoma miss rate (AMR) and polyp miss rate. These studies 13,15,16 found that colonoscopy with AI reduced AMR and polyp miss rate more than routine colonoscopy. The studies 6–13,15,16 concluded that AIC improved the adenoma detection rate and polyp detection rate when compared to conventional colonoscopy. Smaller adenomas (<5 mm) were detected in a significantly higher proportion of subjects in the AI group than in the control group, according to the study^15^. When compared to conventional colonoscopy, one study by Areia *et al.* 2 found lower costs (saving $57 per individual) for lesions detected by AI colonoscopy. This study also reported a higher relative decrease in CRC mortality with AIC by 6% compared to conventional colonoscopy.

The expenses per screened person dropped from $3400 to $3343, saving $57 per person because of AI detection techniques. In other words, AIC reduced the costs of CRC care by an average of 8.2% from $1636 to $1502. The adoption of AI detection during screening colonoscopies prevented an extra 7194 instances of CRC and 2089 deaths connected to it each year, saving the US public at large $290 million per year^2^.

## Discussion

Data extracted from 15 published studies show an increase in the detection of precancerous lesions, such as adenomas, polyps, or carcinomas, using AIC in patients over the age of 18 compared to conventional colonoscopy in this SR. Furthermore, this study shows that AIC reduces AMRs and colorectal neoplasia by 50%^13^,^16^. A decrease in the miss rate of 10 mm flat neoplasia in the proximal and distal colon explains this significant improvement brought about by AIC. Flat neoplasia can be ambiguous and thus missed during a standard colonoscopy. AI used in conjunction with colonoscopy imaging aids in the detection of these less obvious lesions. Biopsy of these lesions, which would otherwise go undetected on conventional colonoscopy, can confirm the histological grade and guide early treatment, improving patient outcomes as well as cost-effectiveness and resource utilization^2^.

Deliwala *et al.* 11, demonstrated that AIC has a higher detection rate for adenomas (77%) [odd ratio (OR): 1.77; 95% CI: 1.570–2.08] and polyps (91%) (OR: 1.91; 95% CI: 1.68–2.16) than standard colonoscopy. Therefore, AIC may help reveal critically underdiagnosed CRC, which can help initiate prompt management.

AI advancements have resulted in recent breakthroughs, such as CADe-assisted colonoscopy, which has demonstrated verifiable success in the identification of adenoma and polyps over high-definition white light colonoscopy^12^. Intelligent retrieval systems can improve the efficacy of colonoscopy screening, lowering the incidence of missed diagnosis^7^,^8^. According to the findings of this study, traditional chromoendoscopy and better mucosal visualization devices have a lower detection rate of malignant lesions than CADe. AI may have a significant impact in terms of quality and efficacy of screening colonoscopy, enhancing a highly sensitive automated detection of adenoma (7.4%) more than CADe (OR: 178; 95% CI: 144–218) representing an objective approach and a superb technique for adenoma detection^9^.

The cost-effectiveness of AIC is worth considering. For example, computer-aided systems for detecting pre-malignant lesions have the potential to save the US government ~$290 million per year^2^. Human errors and blind spots are major contributors to missing lesions during a colonoscopy. AIC allows for more detailed characterization of polyps and adenomas that are difficult to detect with traditional colonoscopy.

Despite the presence of numerous advanced colonoscopy modalities for detecting pathological lesions, these advanced modalities are largely dependent on endoscopist training^16^. Operator dependency is a significant disadvantage because it is impossible to train all endoscopists at the same level^6^. AIC aims to close this gap by utilizing a consistent set of principles that results in the correct classification and identification of lesions using a consistent and organized system^16^.

Several studies have reported a higher polyp and adenoma detection rate when using AIC, but these studies were conducted under ideal conditions. More research is needed to account for these situations because bowel preparation quality can affect endoscopists’ detection of pathological lesions^14^. Becq *et al.* 17 performed a study to evaluate the efficacy of AIC in a real-time setting with variable quality of bowel preparation. Their study found that using AI-assisted tools yielded a high sensitivity of 98.2%^17^. Based on the findings, clinicians can decide whether additional investigations (such as a biopsy) are needed to confirm malignancies and prepare a management plan for discussion^18^. In addition to improved lesion detection, AI-assisted tools evaluate the stimulus in less time to evaluate the stimulus and respond to it^11^,^19^,^20^.

This review included several study designs, predominantly original studies from three widely accepted databases, which allowed us to synthesize an appropriate amount of data required to make direct conclusions on the effectiveness of AIC, which can have broad implications on CRC. The majority of the studies included in this review were RCTs and MAs, demonstrating evidence that does in fact favor AIC when it comes to a comparison of efficacy.

However, more robust studies involving a broader patient population and various stages of bowel prep (poor, adequate, and excellent) are warranted in order to more broadly generalize results. It is also relevant to consider the heterogeneity of statistical tools utilized in the current literature, further studies utilizing standardized statistical tools are warranted to enable more comprehensive comparisons. In addition, there is a scarcity of data stratifying the effectiveness of AIC according to sex, race, as well as its implementation or availability in different socioeconomic classes. This review also prompts future research that can classify and compare different AI-trained algorithms and their impact on early diagnosis of CRC, which further contributes to the current knowledge of AIC and its utility. Although one of the studies included in this review reported better cost-effectiveness of AIC screening compared to conventional colonoscopy, this requires further verification and broader evidence in order to reach a substantial conclusion on cost-effectiveness^21^,^22^. Further large-scale studies on the costs associated with AIC can have implications on its feasibility, access, as well as utility, especially in lower socioeconomic classes. Another limitation encountered in this review was the availability of data on the role of AIC in altering the CRC prognosis and overall patient outcomes.

Continuous research on enhancing screening opens new diagnostic avenues to consider, such as exploring how AI can complement other advances such as capsule colonoscopy, where a camera in tablet form is swallowed orally and is then allowed to pass through the digestive tract. This review, therefore, encourages further retrospective and prospective studies on the application of AIC in CRC patients and its impact on their management plan and survival.

## Conclusion

AI in diagnostic medicine holds enormous promise for improving detection and allowing for earlier management, resulting in better patient outcomes. Recent advancements in AI have been enabled by technologies such as CADe-assisted colonoscopy, which has proven effective in detecting polyps and adenomas during high-definition white light colonoscopy. Polyps and adenomas can be identified using today’s machine-learning-based system, increasing the detection rate over traditional methods. Furthermore, improved diagnostic precision and the AIC offer lower screening costs. However, a lack of data and contradictory evidence obscure its ergonomic and economic benefits. The evidence generated in this SR encourages more large-scale, multicenter research into the cost differences between AIC and conventional colonoscopy, as well as its impact on overall CRC treatment costs, which can ultimately lead to quality improvement in CRC care.

## Ethical approval

Not applicable.

## Consent for publication

Not applicable.

## Sources of funding

None.

## Author contributions

A.M. and A.I.: conceptualization ideas. All authors were involved in the processes of data curation, writing of initial draft, review and editing, and final approval of manuscript.

## Conflicts of interest disclosure

Authors declare no conflict of interest.

## Research registration unique identifying number (UIN)

1. Name of the registry: NA.
2. Unique identifying number or registration ID: NA.
3. Hyperlink to your specific registration (must be publicly accessible and will be checked): NA.

## Guarantor

Wireko Andrew Awuah.

## Data availability statement

No new data generated.
